# Variation in Yield, Chemical Composition and Biological Activities of Essential Oil of Three *Curcuma* Species: A Comparative Evaluation of Hydrodistillation and Solvent-Free Microwave Extraction Methods

**DOI:** 10.3390/molecules28114434

**Published:** 2023-05-30

**Authors:** Swagat Mohanty, Asit Ray, Pradeep Kumar Naik, Ambika Sahoo, Sudipta Jena, Prabhat Kumar Das, Jeetendranath Patnaik, Pratap Chandra Panda, Sanghamitra Nayak

**Affiliations:** 1 Centre for Biotechnology, Siksha ‘O’ Anusandhan (Deemed to be University), Kalinga Nagar, Bhubaneswar 751003, Odisha, India; swagatrude@gmail.com (S.M.); asitray2007@gmail.com (A.R.); ambikasahoo@soa.ac.in (A.S.); jena.sudipta1991@gmail.com (S.J.); prabhatdasnou@gmail.com (P.K.D.); pcpanda2001@yahoo.co.in (P.C.P.); 2 Department of Biotechnology and Bioinformatics, Sambalpur University, Jyoti Vihar, Burla 768018, Odisha, India; pknaik1973@gmail.com; 3 Department of Botany, Sri Krushna Chandra Gajapati Autonomous College, Paralakhemundi 761200, Odisha, India; jeetendranath.patnaik@gmail.com

**Keywords:** *Curcuma* species, hydrodistillation, solvent free microwave extraction, GC-MS, antioxidant activity, anti-tyrosinase activity, anticancer activity

## Abstract

The essential oils of three medicinally important *Curcuma* species (*Curcuma alismatifolia*, *Curcuma aromatica* and *Curcuma xanthorrhiza*) were extracted using conventional hydro-distillation (HD) and solvent free microwave extraction (SFME) methods. The volatile compounds from the rhizome essential oils were subsequently analysed by GC–MS. The isolation of essential oils of each species was carried out following the six principles of green extraction and comparison was made between their chemical composition, antioxidant, anti-tyrosinase and anticancer activities. SFME was found to be more efficient than HD in terms of energy savings, extraction time, oil yield, water consumption and waste production. Though the major compounds of essential oils of both the species were qualitatively similar, there was a significant difference in terms of quantity. The essential oils extracted through HD and SFME methods were dominated by hydrocarbon and oxygenated compounds, respectively. The essential oils of all *Curcuma* species exhibited strong antioxidant activity, where SFME was significantly better than HD with lower IC_50_ values. The anti-tyrosinase and anticancer properties of SFME-extracted oils were relatively better than that of HD. Further, among the three *Curcuma* species, *C. alismatifolia* essential oil showed the highest rates of inhibition in DPPH and ABTS assay, significantly reduced the tyrosinase activity and exhibited significant selective cytotoxicity against MCF7 and PC3 cells. The current results suggested that the SFME method, being advanced, green and fast, could be a better alternative for production of essential oils with better antioxidant, anti-tyrosinase and anticancer activities for application in food, health and cosmetic industries.

## 1. Introduction

Nowadays, there is a growing demand for aromatic plants due to the global resurgence of interest in natural products for health care and therapeutic purposes. The therapeutic properties of herbal and aromatic plants are partially attributed to essential oils. Essential oils, a group of interesting natural products, have gained greater popularity in food, cosmetics, aromatherapy and pharmaceutical industries as an alternative to synthetic chemicals [1,2,3,4]. The essential oils have been found to possess antioxidant, antimicrobial, antiviral, antiaging, anti-inflammatory, anticancer, anti-tyrosinase and many other biological activities [1,2,4].

Variation in volatile oil yield and composition in plants is influenced by their genetic constitution and environmental factors, but according to recent findings, differences in the qualitative and quantitative composition of essential oil are also affected by different extraction methods [5,6,7]. The method of extraction is the primary determinant of the quality of essential oils since poor extraction techniques may result in their degradation and alter the quality of phytochemicals present in aromatic oils. The consequences may include loss of pharmacological compounds, discoloration, off-flavour/odour and physical changes [8]. Therefore, an efficient extraction method is essential for the isolation of phytoconstituents from plants employing optimal conditions and suitable techniques. However, selection of the appropriate extraction method depends on the type and nature of the sample, yield of targeted bioactive compounds and cost of extraction.

*Curcuma*, a genus of the Zingiberaceae family, includes over 100 species of herbaceous rhizomatous perennial plants worldwide, including about 40 species in India [4,9]. It is extensively distributed throughout the tropical and subtropical parts of many continents such as Asia, Africa, and Australia [10]. The species of *Curcuma*, being rich sources of bioactive compounds and credited with antidiabetic, anti-inflammatory, antiviral, antiaging, antimicrobial, antihepatotoxic, antioxidant and anticancer properties, are gaining much attention across the world for use in the formulation of potential novel drugs to treat various disorders [11]. Furthermore, *Curcuma* essential oils have also been reported to improve immunological function, enhance blood circulation, eliminate toxins and stimulate digestion [4]. The rhizomes of *Curcuma* species are rich sources of essential oils, possessing a pleasant aroma and therapeutic potential, and the essential oils of many species are used in traditional and ethnomedicine to treat various diseases. *Curcuma longa* is the most widely used and explored species of the genus *Curcuma.* Some of the underexplored species of the genus are *Curcuma aromatica, Curcuma xanthorrhiza* and *Curcuma alismatifolia.* Rhizomes of *C. aromatica* are used as a carminative, antidote for snakebite, remedy for sprains, hiccoughs, bronchitis, cough, leucoderma and treatment for skin eruptions [12]. Essential oils of *C. aromatica* possess antifungal and antimicrobial properties and are reportedly used for treating the early stages of cervical cancer [13]. Traditional uses of *C. xanthorrhiza* include the treatment of rheumatism, vaginal discharge, liver disease and constipation. It also possesses anti-inflammatory, anticancer, antibacterial, antioxidant, hepatoprotective and other pharmacological properties [14]. Further, *C. alismatifolia* is widely cultivated as an ornamental species for cut flowers and potted plants because of the attractive inflorescence with pink coma bracts and long vase life. It has been claimed to exhibit antioxidant, anti-inflammatory and wound-healing properties [15,16]. The biological activities of *Curcuma* species may be attributed to the presence of non-volatile components and volatile essential oils [17,18,19]. The essential oils of *Curcuma* species are comprised of a wide variety of volatile sesquiterpenes, monoterpenes and other aromatic compounds [4]. Because of the great importance of *Curcuma* essential oils in several applications, various methods of extraction have been developed. Conventional techniques such as hydrodistillation (HD), steam distillation (SD), solvent extraction or supercritical fluid extraction are the most common methods for extracting volatile oils from the rhizomes of *Curcuma* species. However, some of the traditional extraction methods have low extraction efficiency, require relatively more time and water input, and there is possibility of thermal degradation or chemical modifications of essential oil constituents [20,21,22,23]. To overcome these issues, solvent free microwave extraction (SFME) method has recently been developed that is known to be a reliable and efficient method to enhance the extraction of phytochemicals from various matrices without much use of aqueous or organic solvent [24]. Significant findings have been reported in various *Curcuma* species, where SFME-extracted oils outperformed HD-extracted oils in terms of yield and extraction time [25]. This green extraction technique involves microwave radiation that generates controlled heat, inducing a pressure gradient where plant metabolites are released by diffusion, breaking and rupturing of cell walls and tissues. The rising temperature causes the vibration of water and polar molecules, disrupting cells and matrices with the release of volatile active compounds by azeotropic distillation [26]. The advantages of SFME over hydro/steam distillation include more effective heating, faster energy transfer, timesaving, lower operating costs and an environmentally friendly process for extracting essential oils from plants [2,27,28]. Due to the controlled temperature and regulated microwave energy, SFME also prevents damage to thermosensitive compounds. The range of extraction parameters such as solvent ratio, microwave power intensity and irradiation period vary depending on the sample, which is mostly employed based on preliminary studies. To date, *C. longa, C. aeruginosa, C. xanthorrhiza* and *C. amada* are among the few *Curcuma* species that have been studied for microwave extraction of essential oil [25,29,30,31]. With respect to biological activities of *Curcuma* essential oils obtained through different extraction methods, except for *C. longa*, the works performed so far on other species are at the elementary level [4]. Thus, the present study was aimed at evaluating the oil yield and chemical composition of rhizome essential oils of *C. alismatifolia* and *C. aromatica* using conventional hydrodistillation and solvent-free microwave extraction methods for the first time. Furthermore, the essential oils of three target species obtained through two different extraction methods were evaluated for their antioxidant, anti-tyrosinase and anticancer activities.

## 2. Results and Discussion

### 2.1. Determination of Extraction Parameters and Essential Oil Yield

**Figure 1:**
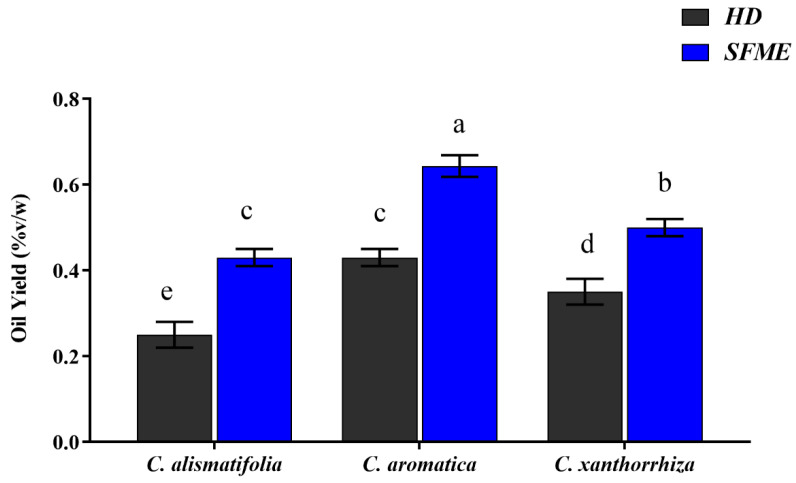
Essential oil yield (%*v*/*w*) of the studied *Curcuma* species obtained by HD and SFME. Oil yield values with distinct superscript letters (a–e) differ significantly at *p* < 0.05 (Tukey test).

Mature and thick rhizomes were selected from the harvested *Curcuma* species to extract their essential oils using HD and SFME methods. The essential oils had a pleasant aroma and were liquid at room temperature. The extraction time, energy consumption, colour of volatile oil and hydration capacity of the two extraction methods are shown in Table 1. A wattmeter was used to measure the power consumption at the entrance of the microwave generator and the electrical heater power source. Figure 1 represents a comparative analysis of essential oil yield of the studied species, which varied in the range of 0.25% and 0.68% (*v*/*w*) on dry weight basis, signifying statistical differences at *p* < 0.05 (Tukey test). Among the three species, the maximum oil yield was recorded for *C. aromatica* (SFME-0.68 ± 0.05%, HD-0.43 ± 0.04%) followed by *C. xanthorrhiza* (SFME-0.52 ± 0.04%, HD-0.35 ± 0.02%) and *C. alismatifolia* (SFME-0.43 ± 0.03%, HD-0.25 ± 0.02%). In *C. alismatifolia*, the oil yield (0.25%) obtained in the present study by the hydrodistillation method was relatively higher than the yield (0.12%) reported by Theanphong et al. [32,33]. Similarly, the oil yield of *C. aromatica* (0.43%) and *C. xanthorrrhiza* (0.35%) obtained through hydrodistillation was relatively less than the oil yields reported in few previous studies [34,35,36]. In comparison to HD, SFME yielded higher amounts of oil in all the species, which might be due to its efficient and homogenous extraction technique, which provides controlled temperature and microwave energy to the sample matrix, as a result of which cells are ruptured by internal superheating, thus facilitating better diffusion of constituents from the matrix and thereby enhancing the recovery of essential oil [37,38]. Several plant species have been found to produce higher amounts of essential oils using a microwave extraction technique [20,29,37]. In an effort to determine the best extraction method, HD and SFME were compared based on the six green extraction principles proposed by Chemat et al. [39]. The analysis was carried out based on raw materials, oil yield, operating duration, waste generation, water and energy consumption.

**Figure 2:**
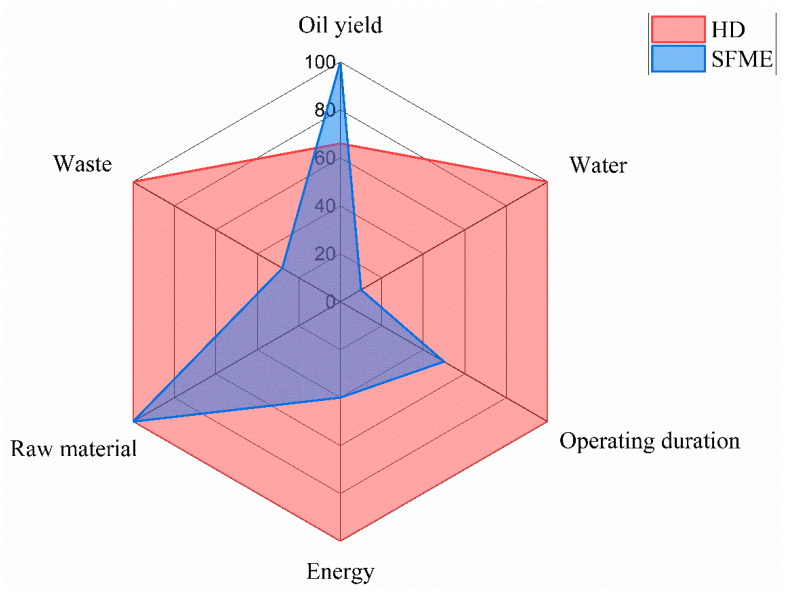
Hydrodistillation (HD) and solvent-free microwave extraction (SFME) classified according to the six green extraction principles. The graph represents the percentage equivalents of each principle’s value.

In Figure 2, the HD and SFME procedures are graphically represented and categorized in accordance with the six green extraction principles. The percentage equivalents of each principle’s values were converted and presented on the graph. The advantages of SFME over HD for the extraction of *Curcuma* essential oil is that it uses less energy, produces less waste, uses less solvent and gives a higher yield over a shorter extraction period.

### 2.2. Comparison of Chemical Composition Obtained by HD and SFME

The identification of compounds of essential oils obtained from both extraction methods was performed using GC-MS analysis; the compounds were classified according to their order of elution on Elite-5 MS column and are presented in Table 2. A total of 85 compounds were identified constituting 90.76% to 94.97% of the total essential oils in the three *Curcuma* species. In *C. alismatifolia*, 50 compounds were identified in essential oils obtained through HD and SFME methods accounting for 94.02% and 94.84% of the total oils, respectively. Similarly, in *C. aromatica* essential oil, 49 compounds could be identified by HD and SFME techniques which constituted 93.12% and 94.97% of the total oil, respectively. The essential oil of *C. xanthorrhiza* had 48 identified chemical constituents. This constitutes 90.76% of the total essential oils isolated through the HD method and 93.82% via the SFME method.

**Table 2:** Chemical composition of essential oils extracted by hydrodistillation (HD) and solvent-free microwave extraction (SFME) from three *Curcuma* species.

Sl No.	RI_exp_ ^i^	RI_lit_ ^ii^	Compound ^iii^	Peak Area %					
				*Curcuma alismatifolia*		*Curcuma aromatica*		*Curcuma xanthorrhiza*	
				HD	SFME	HD	SFME	HD	SFME
1	926	926	Tricyclene	0.13 ± 0.02 ^a^	0.10 ± 0.03 ^b^	0.13 ± 0.04 ^a^	0.14 ± 0.05 ^a^	-	-
2	930	930	*α*-Thujene	1.32 ± 0.14 ^a^	0.23 ± 0.05 ^cd^	1.15 ± 0.0 ^b^	0.12 ± 0.02 ^de^	0.27 ± 0.06 ^c^	0.07 ± 0.01 ^e^
3	946	939	*α*-Pinene	3.18 ± 0.27 ^a^	1.40 ± 0.18 ^b^	0.08 ± 0.02 ^a^	0.10 ± 0.03 ^e^	1.03 ± 0.14 ^c^	0.74 ± 0.14 ^d^
4	950	954	Camphene	0.88 ± 0.12 ^c^	0.91 ± 0.13 ^c^	3.28 ± 0.31 ^a^	1.61 ± 0.25 ^b^	0.35 ± 0.09 ^e^	0.78 ± 0.10 ^d^
5	974	975	Sabinene	0.31 ± 0.04 ^c^	0.50 ± 0.11 ^b^	0.82 ± 0.15 ^a^	0.10 ± 0.05 ^d^	-	-
6	984	979	*β*-Pinene	4.96 ± 0.32 ^a^	2.86 ± 0.24 ^b^	0.88 ± 0.14 ^d^	0.08 ± 0.05 ^f^	0.25 ± 0.09 ^e^	1.73 ± 0.22 ^c^
7	1003	990	Myrcene	0.07 ± 0.01 ^a^	0.08 ± 0.02 ^a^	-	-	-	-
8	1006	1011	*δ-3*-Carene	1.58 ± 0.14 ^a^	0.09 ± 0.03 ^b^	-	-	-	-
9	1020	1024	*p*-Cymene	0.15 ± 0.03 ^a^	0.13 ± 0.02 ^a^	-	-	-	-
10	1025	1029	Limonene	1.00 ± 0.15 ^b^	1.67 ± 0.18 ^a^	1.80 ± 0.21 ^a^	0.40 ± 0.08 ^c^	0.25 ± 0.07 ^cd^	0.17 ± 0.02 ^d^
11	1029	1031	1,8-cineole	6.47 ± 0.78 ^c^	16.60 ± 1.45 ^a^	13.02 ± 1.32 ^b^	7.55 ± 0.88 ^c^	0.80 ± 0.18 ^d^	0.81 ± 0.17 ^d^
12	1051	1059	*γ*-Terpinene	-	-	0.18 ± 0.04 ^a^	0.04 ± 0.01 ^b^	-	-
13	1081	1088	Terpinolene	0.14 ± 0.03 ^a^	0.10 ± 0.01 ^c^	0.27 ± 0.04 ^b^	0.29 ± 0.07 ^a^	0.45 ± 0.05 ^a^	0.17 ± 0.02 ^c^
14	1085	1090	2-Nonanone	0.19 ± 0.05 ^a^	0.21 ± 0.05 ^a^	-	-	-	-
15	1097	1096	Linalool	1.93 ± 0.18 ^b^	0.73 ± 0.09 ^c^	2.87 ± 0.47 ^a^	2.99 ± 0.37 ^a^	0.40 ± 0.09 ^d^	0.78 ± 0.11 ^c^
16	1113	1114	*trans*-Thujone	0.10 ± 0.02 ^a^	0.12 ± 0.04	-	-	-	-
17	1143	1146	Camphor	3.78 ± 0.74 ^d^	9.61 ± 1.24 ^b^	8.45 ± 1.22 ^c^	14.04 ± 2.01 ^a^	3.33 ± 0.58 ^e^	2.71 ± 0.42 ^f^
18	1157	1160	Isoborneol	-	-	-	-	0.14 ± 0.05 ^a^	0.68 ± 0.15 ^a^
19	1159	1166	*δ*-Terpineol	1.50 ± 0.24 ^a^	0.12 ± 0.05 ^b^	-	-	-	-
20	1160	1168	3-Thujanol	0.29 ± 0.07 ^a^	4.35 ± 0.53 ^a^	-	-	-	-
21	1160	1169	Borneol	-	-	2.90 ± 0.14 ^b^	6.04 ± 0.41 ^a^	0.15 ± 0.02 ^d^	0.56 ± 0.07 ^c^
22	1174	1177	Terpinen-4-ol	0.57 ± 0.19 ^b^	0.35 ± 0.14 ^d^	0.80 ± 0.22 ^a^	0.43 ± 0.14 ^c^	0.08 ± 0.01 ^f^	0.26 ± 0.08 ^e^
23	1192	1188	*α*-Terpineol	0.19 ± 0.04 ^d^	0.60 ± 0.17 ^c^	0.90 ± 0.10 ^b^	1.02 ± 0.11 ^a^	0.07 ± 0.01 ^e^	0.61 ± 0.04 ^c^
24	1272	1267	Geranial	0.43 ± 0.06 ^b^	0.63 ± 0.10 ^a^	-	-	-	-
25	1281	1285	Isobornyl acetate	-	-	0.07 ± 0.05 ^a^	0.05 ± 0.01 ^a^	-	-
26	1287	1285	Bornyl acetate	0.12 ± 0.08 ^a^	0.09 ± 0.02 ^a^	-	-	-	-
27	1290	1294	2-Undecanone	0.18 ± 0.03 ^a^	0.10 ± 0.04 ^a^	-	-	-	-
28	1328	1338	*δ*-Elemene	0.12 ± 0.03 ^d^	0.18 ± 0.05 ^c^	0.61 ± 0.09 ^a^	0.07 ± 0.01 ^e^	0.18 ± 0.09 ^a^	0.45 ± 0.08 ^b^
29	1371	1351	*α*-Cubebene	-	-	-	-	1.88 ± 0.29 ^a^	1.89 ± 0.34 ^a^
30	1375	1375	*α*-Ylangene	0.17 ± 0.04 ^a^	0.24 ± 0.06 ^b^	-	-	-	-
31	1391	1376	*α*-Copaene	-	-	0.44 ± 0.08 ^b^	1.13 ± 0.12 ^a^	-	-
32	1395	1390	*β*-Elemene	1.30 ± 0.14 ^d^	3.34 ± 0.21 ^b^	4.03 ± 0.19 ^a^	1.85 ± 0.14 ^c^	-	-
33	1409	1408	*(Z)*-Caryophyllene	-	-	0.21 ± 0.09 ^a^	0.36 ± 0.09 ^c^	1.84 ± 0.21 ^a^	0.54 ± 0.10 ^b^
34	1411	1411	*α*-Cedrene	1.55 ± 0.24 ^b^	1.97 ± 0.25 ^a^	0.09 ± 0.03 ^a^	0.07 ± 0.01 ^c^	-	-
35	1426	1419	*β*-Caryophyllene	0.26 ± 0.05 ^c^	0.36 ± 0.08 ^b^	1.69 ± 0.22 ^a^	0.26 ± 0.07 ^a^	-	-
36	1427	1434	*trans-α*-Bergamotene			0.27 ± 0.05 ^a^	0.23 ± 0.07 ^a^	0.12 ± 0.04 ^b^	0.08 ± 0.02 ^c^
37	1439	1436	*γ*-Elemene	0.24 ± 0.05 ^a^	0.05 ± 0.01 ^c^	-	-	0.20 ± 0.08 ^b^	0.18 ± 0.02 ^b^
38	1444	1439	*α*-Guaiene	-	-	0.45 ± 0.18 ^a^	0.22 ± 0.10 ^a^	-	-
39	1444	1441	Aromadendrene	0.63 ± 0.21 ^a^	0.19 ± 0.08 ^b^	-	-	-	-
40	1446	1442	*(Z)-β*-Farnesene	-	-	0.46 ± 0.04 ^a^	0.18 ± 0.08 ^a^	0.23 ± 0.07 ^b^	0.08 ± 0.04 ^c^
41	1463	1456	*(E)-β*-Farnesene	-	-	-	-	0.25 ± 0.03 ^b^	0.87 ± 0.09 ^a^
42	1465	1460	*allo*-Aromadendrene	-	-	-	-	0.27 ± 0.04 ^a^	0.22 ± 0.08
43	1470	1466	*cis*-Muurola-4(14),5-diene	-	-	-	-	0.63 ± 0.14 ^a^	0.11 ± 0.04
44	1478	1477	*γ*-Gurjunene	0.05 ± 0.01	0.64 ± 0.21 ^b^	-	-	1.63 ± 0.30 ^a^	0.54 ± 0.14 ^c^
45	1479	1480	*ar*-Curcumene	0.77 ± 0.12	1.07 ± 0.14 ^d^	1.85 ± 0.24 ^c^	0.77 ± 0.15 ^e^	3.27 ± 0.66 ^a^	2.96 ± 0.46 ^b^
46	1484	1481	Germacrene D	-	-	0.63 ± 0.14 ^a^	0.04 ± 0.01 ^b^	-	-
47	1492	1490	*β*-Selinene	-	-	0.25 ± 0.07 ^b^	0.21 ± 0.04	0.18 ± 0.08 ^c^	0.42 ± 0.12 ^a^
48	1492	1493	*cis-β*-Guaiene	-	-	-	-	0.13 ± 0.02 ^b^	0.33 ± 0.11 ^a^
49	1496	1493	*α*-Zingiberene	1.92 ± 0.36 ^a^	0.65 ± 0.25 ^d^	0.89 ± 0.10 ^b^	0.08 ± 0.04	0.71 ± 0.09 ^c^	0.56 ± 0.08 ^e^
50	1503	1499	Curzerene	0.11 ± 0.04	0.19 ± 0.05 ^d^	4.18 ± 0.74 ^a^	2.02 ± 0.34 ^b^	0.21 ± 0.11	0.48 ± 0.08 ^c^
51	1505	1500	*β*-Himachalene	0.50 ± 0.09 ^a^	0.06 ± 0.04	0.46 ± 0.1 ^b^	0.09 ± 0.02	-	-
52	1507	1505	*β*-Bisabolene	-	-	0.75 ± 0.09 ^a^	0.13 ± 0.04	-	-
53	1509	1515	*β*-Curcumene	0.12 ± 0.02 ^b^	0.19 ± 0.08 ^a^	-	-	-	-
54	1538	1522	*β*-Sesquiphellandrene	-	-	2.16 ± 0.05 ^a^	0.04 ± 0.01 ^c^	0.10 ± 0.02 ^b^	0.08 ± 0.03
55	1548	1546	Selina-3,7(11)-diene	0.54 ± 0.09 ^a^	0.18 ± 0.07	-	-	0.07 ± 0.01 ^b^	0.09 ± 0.02
56	1569	1549	Elemol	-	-	0.19 ± 0.07	0.34 ± 0.05 ^c^	0.73 ± 0.1 ^a^	0.65 ± 0.08 ^b^
57	1572	1561	Germacrene B	0.26 ± 0.05 ^b^	0.52 ± 0.09 ^a^	-	-	-	-
58	1578	1563	*(E)*-Nerolidol	-		0.33 ± 0.07 ^a^	0.09 ± 0.02	0.12 ± 0.01 ^b^	0.06 ± 0.01 ^c^
59	1580	1575	Germacrene D-4-ol	0.12 ± 0.04	0.37 ± 0.08 ^a^	-	-	0.27 ± 0.10 ^b^	0.15 ± 0.07 ^c^
60	1581	1578	Spathulenol	-	-	0.09 ± 0.02	0.07 ± 0.01 ^a^	-	-
61	1586	1583	Caryophyllene oxide	-		0.29 ± 0.05 ^a^	0.05 ± 0.01 ^d^	0.20 ± 0.04 ^b^	0.10 ± 0.02 ^c^
62	1594	1606	Curzerenone	40.60 ± 2.46 ^c^	26.47 ± 1.88 ^d^	2.22 ± 0.47 ^f^	7.14 ± 1.88 ^e^	56.34 ± 3.62 ^b^	62.81 ± 4.12 ^a^
63	1605	1607	*5-epi-7-epi-α*-Eudesmol	0.11 ± 0.03	0.08 ± 0.02 ^a^	-	-	-	-
64	1607	1608	*β*-Atlantol	0.78 ± 0.14 ^a^	0.18 ± 0.02 ^b^	-	-	-	-
65	1611	1608	Humulene epoxide II	-	-	0.76 ± 0.14 ^a^	0.09 ± 0.02 ^d^	0.49 ± 0.09 ^c^	0.65 ± 0.13 ^b^
66	1623	1619	*1,10-di-epi*-Cubenol	-	-	0.22 ± 0.05 ^a^	0.13 ± 0.03 ^b^	0.13 ± 0.04 ^b^	0.06 ± 0.01 ^c^
67	1631	1631	Muurola-4,10(14)-dien-1-β-ol	0.27 ± 0.04 ^a^	0.09 ± 0.02 ^b^	-	-	-	-
68	1632	1632	*γ*-Eudesmol	-	-	-	-	0.31 ± 0.10 ^a^	0.07 ± 0.03 ^b^
69	1646	1646	Cubenol	0.51 ± 0.09 ^b^	0.17 ± 0.04 ^b^	1.29 ± 0.24 ^a^	0.25 ± 0.08 ^b^	1.28 ± 0.44 ^a^	0.63 ± 0.18 ^b^
70	1648	1650	*β*-Eudesmol	-	-	0.20 ± 0.05	0.16 ± 0.04 ^a^	-	-
71	1654	1653	*α*-Eudesmol	0.32 ± 0.10 ^a^	0.12 ± 0.02 ^b^	-	-	0.11 ± 007 ^b^	0.12 ± 0.06
72	1659	1669	*ar*-Turmerone	-	-	-	-	0.17 ± 0.08 ^c^	0.29 ± 0.07 ^a^
73	1667	1675	*β*-Bisabolol	0.17 ± 0.03 ^a^	0.12 ± 0.02 ^b^	-	-	0.09 ± 0.04 ^c^	0.07 ± 0.03 ^c^
74	1670	1677	*(Z)*-Nerolidyl acetate	-	-	0.12 ± 0.05	0.05 ± 0.01 ^a^	-	-
75	1684	1693	Germacrone	8.63 ± 1.21 ^c^	12.35 ± 2.04 ^b^	6.69 ± 1.78 ^d^	16.44 ± 3.03 ^a^	4.22 ± 0.78 ^f^	4.34 ± 1.0 ^e^
76	1703	1701	Curlone (*β*-Turmerone)	-	-	14.19 ± 2.26 ^a^	12.31 ± 1.55 ^b^	-	-
77	1715	1718	Curcuphenol	-	-	0.61 ± 0.08 ^a^	0.14 ± 0.01 ^b^	-	-
78	1716	1718	*(Z)-α*-Atlantone	0.16 ± 0.03 ^b^	0.45 ± 0.10 ^a^	-	-	-	-
79	1720	1731	Chamzulene	-	-	-	-	0.14 ± 0.04	0.11 ± 0.02 ^a^
80	1722	1733	Zerumbone	-	-	-	-	0.15 ± 0.04	0.19 ± 0.03 ^a^
81	1738	1734	Curcumenol	4.06 ± 0.45 ^a^	1.87 ± 0.98 ^b^	1.64 ± 0.55 ^d^	1.69 ± 0.25 ^c^	-	-
82	1754	1747	Neocurdione			0.51 ± 0.12 ^a^	0.23 ± 0.03 ^b^	-	-
83	1771	1753	Xanthorrhizol	0.30 ± 0.06 ^f^	1.19 ± 0.27 ^e^	6.76 ± 1.23 ^b^	13.07 ± 1.96 ^a^	6.23 ± 1.09 ^c^	2.84 ± 0.81 ^d^
84	1783	1778	*α-(E)*-Atlantone	-	-	-	-	1.02 ± 0.22 ^a^	0.40 ± 0.08 ^b^
85	1789	1784	*γ*-Eudesmol acetate	-	-	-	-	0.04 ± 0.01	0.35 ± 0.07 ^a^
Monoterpene hydrocarbons				15.64 ± 0.57	8.79 ± 0.23	11.45 ± 0.35	5.88 ± 0.12	3.01 ± 0.66	4.44 ± 0.11
Oxygenated monoterpenes				13.53 ± 0.84	33.02 ± 0.87	30.32 ± 0.85	31.13 ± 1.03	4.43 ± 0.28	5.62 ± 0.18
Sesquiterpene hydrocarbons				8.18 ± 0.44	9.46 ± 0.66	15.26 ± 1.12	5.72 ± 0.18	11.43 ± 0.64	9.78 ± 0.38
Oxygenated sesquiterpenes				55.76 ± 1.25	43.08 ± 1.02	36.09 ± 1.56	52.24 ± 1.24	71.78 ± 1.88	73.42 ± 0.56
Others				0.91 ± 0.11	0.49 ± 0.08	-	-	0.11 ± 0.01	0.56 ± 0.09
Total				94.02 ± 0.94	94.84 ± 1.64	93.12 ± 0.66	94.97 ± 0.76	90.76 ± 0.87	93.82 ± 0.35

Data are provided as mean ± S.D (*n* = 3). According to the Tukey test, means in the same row that are denoted by distinct superscript letters are significantly different at (*p* < 0.05), - not detected, ^i^ Calculated retention indices (RI) for the Elite-5 MS column in comparison to the C_8_–C_20_ n-alkane series, ^ii^ Retention indices from literature [40], ^iii^ Compound listed in the order of elution on Elite-5 MS capillary column (30 m × 0.25 mm × 0.25 μm column thickness).

Overall, the chemical makeup of the essential oils of the three studied *Curcuma* species was dominated by oxygenated sesquiterpenes (36.09 to 73.42%) followed by oxygenated monoterpenes (4.43 to 31.13%), where both the chemical groups were present in substantially higher amounts in oils extracted through SFME method compared to HD. The amount of sesquiterpene hydrocarbons (5.72 to 15.26%) and monoterpene hydrocarbons (3.01 to 15.64%) in the essential oils varied considerably between the two extraction methods. Table 2 shows that HD is more efficient in isolating hydrocarbon molecules, whereas SFME is more effective in isolating most of the oxygenated compounds. Djouahri et al. [37] made a similar observation while extracting *Tetraclinis articulata* essential oil using hydrodistillation and microwave-assisted extraction. Significant differences were observed in the quantity of major compounds between the two methods which might be due to a reduction in thermal and hydrolytic reactions compared with hydrodistillation, which utilizes large amounts of water and energy [41]. Similar results were obtained in *C. longa* essential oil that reported differences in major compounds extracted by a microwave-assisted technique [42]. The variations in the main constituents had no significant effect on the bioactivities of the essential oils.

**Figure 3:**
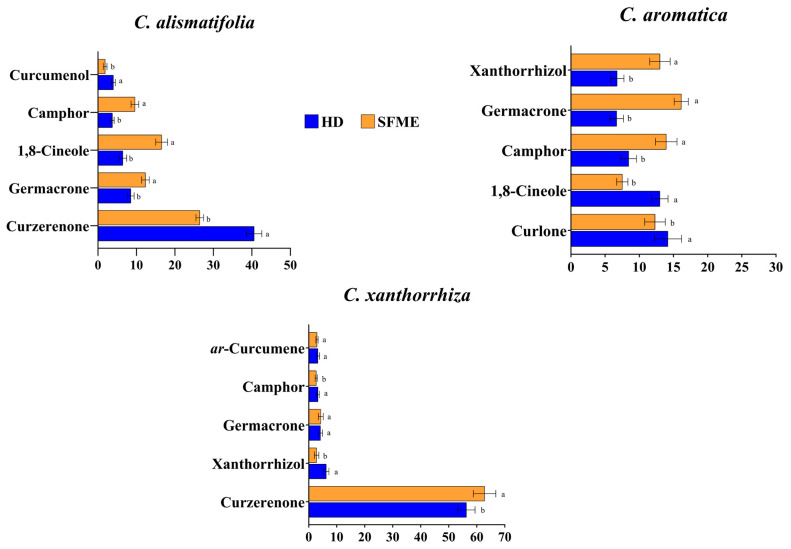
Percentage composition of major compounds in *C. alismatifolia*, *C. aromatica* and *C. xanthorrhiza* essential oils obtained using HD and SFME methods. Different letters denote a significant difference in the mean value of constituents at (*p* < 0.05).

Figure 3 shows the percentage composition of the principal components of essential oils of the three *Curcuma* species using both the techniques. The major compounds identified in the essential oil of *C. alismatifolia* are curzerenone (40.6% vs. 26.47% for HD vs. SFME), germacrone (8.63% vs. 12.35% for HD vs. SFME), 1,8 cineole (6.47% vs. 16.6% for HD vs. SFME) and camphor (3.78 % vs. 9.61% for HD vs. SFME). Similarly, essential oil of *C. aromatica* contained curlone (*β*-turmerone) (14.19% vs. 12.31% for HD vs. SFME), germacrone (6.69% vs. 16.44% for HD vs. SFME), 1,8 cineole (13.02% vs. 7.6% for HD vs. SFME), camphor (8.45% vs. 14.04% for HD vs. SFME) and xanthorrhizol (6.76% vs. 13.07% for HD vs. SFME), whereas curzerenone (56.34% vs. 62.81% for HD vs. SFME), xanthorrhizol (6.23% vs. 2.84% for HD vs. SFME), germacrone (4.21% vs. 4.44% for HD vs. SFME) and camphor (3.33% vs. 2.71% for HD vs. SFME) were detected in *C. xanthorrhiza* essential oil. Very few reports are available on the chemical composition of *C. alismatifolia* rhizome essential oil that revealed *β*-curcumene and xanthorrhizol as its major constituents [32,33]. The current study identified a new curzerenone-rich essential oil bearing chemotype of *C. alismatifolia* extracted using both HD and SFME methods. The chemical composition of *C. aromatica* essential oil now reported is consistent with the earlier reports, where curlone (*β*-turmerone), 1,8 cineole, germacrone, and xanthorrhizol have been found to be the major constituents. Similarly, curzerenone, xanthorrhizol, germacrone, and camphor were detected as predominant components in the essential oil of *C. xanthorrhiza* [14,35,43,44,45,46]. Furthermore, compounds such as *ar*-curcumene, xanthorrhizol and *β*-curcumene, reported as major compounds in earlier studies, were present in trace amounts in the present investigation [32,47,48]. No qualitative variations in essential oils were observed between the extraction methods of each species as the compounds detected either by HD or SFME were the same. Fiorini et al. [20] and Araujo et al. [21] also observed no qualitative differences between essential oils of *Cannabis sativa* and *Thymus mastichina* extracted from the hydrodistillation and microwave extraction methods, respectively. For the first time, the present study utilized HD and SFME methods to conduct a qualitative and quantitative analyses of the yield and chemical composition of the essential oils of *C. alismatifolia*, *C. aromatica* and *C. xanthorrhiza.*

### 2.3. Antioxidant Activity

**Figure 4:**
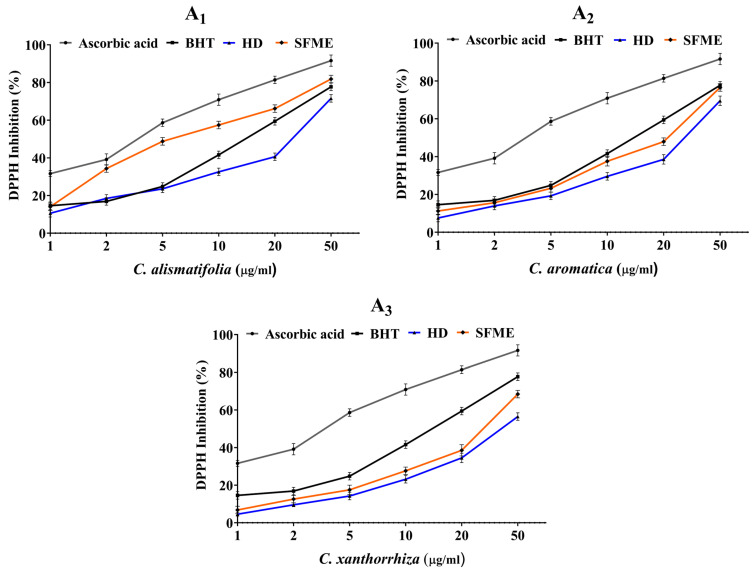
Antioxidant activity of *Curcuma* essential oils obtained by HD and SFME methods along with standard antioxidants using (A) DPPH assay. Data are represented as mean ± S.D. (*n* = 3).

**Figure 5:**
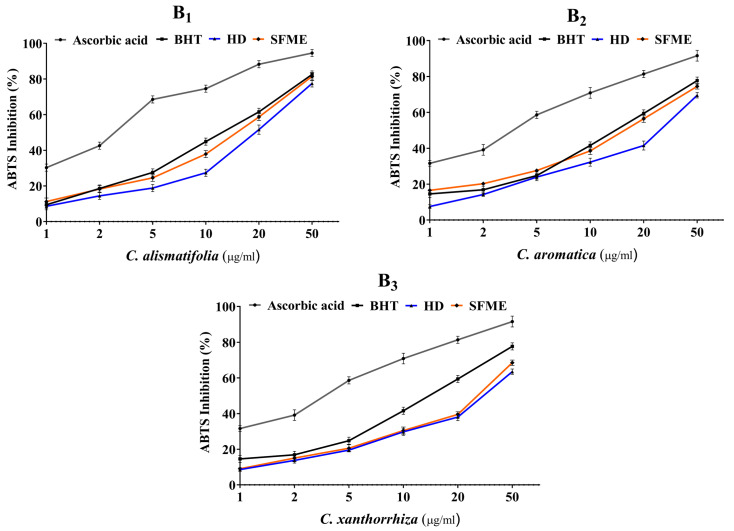
Antioxidant activity of *Curcuma* essential oils obtained by HD and SFME along with standard antioxidants using (B) ABTS assay. Data are represented as mean ± S.D. (*n* = 3).

The in vitro antioxidative capacity of three *Curcuma* essential oils extracted via HD and SFME was studied using DPPH and ABTS radical scavenging assays. The DPPH and ABTS radicals are prominent substrates for rapid evaluation of antioxidant activity due to their radical stability and efficiency. Butylated hydroxytoluene and ascorbic acid were taken as the standard positive controls for both the assays. Several concentrations ranging from 1–50 μg/mL were used to analyse the percentage of inhibition as shown in Figure 4 and Figure 5. Their IC_50_ values were also calculated. In DPPH assay, the *Curcuma* essential oils exhibited moderate scavenging activity compared with BHT and ascorbic acid, with an IC_50_ value of 15.6 ± 0.14 μg/mL and 4.2 ± 0.08 μg/mL, respectively. *C. alismatifolia* essential oil had the maximum antioxidant activity with an IC_50_ value of 28.2 ± 0.19 μg/mL for HD and 19.1 ± 0.16 μg/mL for SFME followed by *C. aromatica* (36.3 ± 0.21 μg/mL vs. 23.1 ± 0.18 μg/mL for HD vs. SFME) and *C. xanthorrhiza* (45.2 ± 0.24 μg/mL vs. 36.8 ± 0.14 μg/mL for HD vs. SFME). Similarly, *Curcuma* essential oils revealed potent scavenging activity in the ABTS assay compared to BHT and ascorbic acid with an IC_50_ value of 13.6 ± 0.12 μg/mL and 3.1 ± 0.06 μg/mL, respectively. In this assay, *C. alismatifolia* essential oil showed the maximum scavenging capacity with an IC_50_ value of 19.3 ± 0.22 μg/mL for HD and 15.24 ± 0.17 μg/mL for SFME followed by *C. aromatica* (28.3 ± 0.27 μg/mL vs. 17.4 ± 0.18 μg/mL for HD vs. SFME) and *C. xanthorrhiza* (36.7 ± 0.22 μg/mL vs. 29.4 ± 0.17 μg/mL for HD vs. SFME). The current research revealed that *C. alismatifolia* essential oil had the highest antioxidant capacity, followed by *C. aromatica* and *C. xanthorrhiza* in both assays.

Our findings are in conformity with those of previously published reports that *C. alismatifolia* extracts have strong antioxidant capacity and good reducing power in which the standard ascorbic acid had an IC_50_ value of 3.76 µg/mL [15,49]. Rhizome essential oils of *C. aromatica* and *C. xanthorrhiza* are also reported to possess considerable antioxidant activity in both the radical scavenging assays and β-carotene bleaching tests as well. Trolox C, ascorbic acid and butylated hydroxyanisole were used as the standard references in these studies, and had an IC_50_ value of 8.82, 7 and 18 µg/mL, respectively [35,50,51,52,53]. The high antioxidant effect of rhizome essential oils might be due to the presence of major compounds such as 1,8 cineole, xanthorrhizol, germacrone, curzerenone and minor constituents including terpinolene and myrcene, which are considered potential antioxidant molecules [43,54]. *Curcuma* species are extensively used as therapeutic agents for disorders caused by free radicals [52] and an attempt has been made in the current study to validate the antioxidant potential of essential oil extracted via HD and SFME methods. The essential oil extracted using SFME was found to be potent scavenger as it inhibited more free radicals than HD at lower concentrations and exhibited low IC_50_ value in all *Curcuma* species. The wide range of IC_50_ values observed between both extraction methods concluded that the antioxidant property of SFME-extracted oil is significantly better than oil extracted through HD. The difference in the results of the antioxidant assay could be due to several factors including temperature and extraction time. Prolonged extraction time and high temperature might be responsible for the breakdown of antioxidant molecules [55]. As previously reported, *C. longa* essential oil possessed higher antioxidant properties in microwave-assisted extraction than the conventional Soxhlet method [29]. Similar findings were also observed in essential oils of several plant species [2,34,50].

It seems possible that a change in the concentration of any specific molecule could play a significant role in expressing the antioxidant and biological properties of the substance. The high antioxidant activity of SFME-derived essential oils might be attributed to the presence of a greater concentrations of oxygenated compounds in *C. alismatifolia* (69.29% vs 76.1% for HD vs. SFME), *C. aromatica* (66.41% vs 83.37% for HD vs. SFME) and *C. xanthorrhiza* (76.01% vs 79.04% for HD vs. SFME), respectively. The present finding is in agreement with the observations of Berga-Zougali et al. [56] from France, who employed HD and SFME methods to characterize *Myrtus communis* essential oil using GC-MS and assessed their bioactivities.

### 2.4. Anti-Tyrosinase Activity

**Figure 6:**
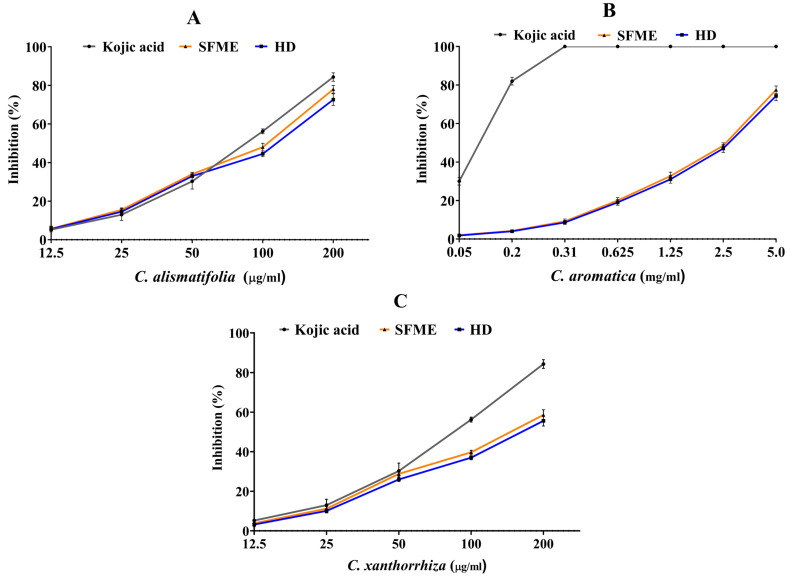
Anti-tyrosinase activity of *Curcuma* essential oils obtained by HD and SFME methods. Figure shows percent inhibition of mushroom tyrosinase enzyme by Kojic acid and (**A**) *C. alismatifolia,* (**B**) *C. aromatica* and (**C**) *C. xanthorrhiza* essential oils. Data are represented as mean ± SD (*n* = 3).

Tyrosinase is a vital enzyme that can accelerate melanin production and enzymic browning caused by bacteria, fungi, plants, insects, and ultraviolet radiation. Free radicals serve a major part in melanin biosynthesis that can cause skin pigmentation, freckles and age spots in humans [57]. The in vitro tyrosinase inhibitory activity of three *Curcuma* essential oils extracted using HD and SFME methods was studied using a spectrophotometric assay. The results indicate a significant percentage inhibition of mushroom tyrosinase enzyme with treatment of essential oil samples and kojic acid at different concentrations (Figure 6). The highest rate of inhibition was shown by *C. alismatifolia* essential oil having an IC_50_ value of 119.1 ± 1.89 μg/mL for HD and 112.4 ± 1.62 μg/mL for SFME, followed by *C. xanthorrhiza* (157.5 ± 2.27 μg/mL vs. 151.4 ± 0.18 μg/mL for HD vs. SFME) and *C. aromatica* (2.71 ± 0.23 mg/mL vs. 2.69 ± 0.22 mg/mL for HD vs. SFME). Kojic acid is a natural tyrosinase inhibitor that inactivates the enzyme by chelating copper and inhibiting tautomerization of dopachrome to 5,6-dihydroxyindole-2-carboxylic acid [58] and was used as a positive control in the present experiment. Though the efficacy of three *Curcuma* essential oils was lower than the standard kojic acid with an IC_50_ value of 82.84 ± 0.12 μg/mL, they can be considered potential tyrosinase inhibitors. The antityrosinase property of the essential oils might be attributed to the presence of specific compounds such as 1,8-cineole, α-pinene, α-terpineol, xanthorrhizol, camphor, which are known enzyme inhibitors [59,60,61]. Different solvent extracts of the rhizomes of *C. aromatica, C. xanthorrhiza, C. longa, C. aeruginosa* and *C. amada* have been investigated in recent times and some important findings have been reported. These studies employed kojic acid as the positive control that showed an IC_50_ value of 0.01 mg/mL [62]. To date, there are no reports on the enzyme-inhibitory activity of *C. alismatifolia* essential oil. Extensive studies on tyrosinase inhibitors in *C. longa* have been made at in silico, in vitro and in vivo levels [63,64,65,66], whereas the remaining species have only been worked on a basic level. Thus, the present observations about the three *Curcuma* species might improve our knowledge on the anti-tyrosinase activity of different species of the genus *Curcuma* and lead to the development of plant-based skin whitening cosmetics.

In *C. alismatifolia* and *C. xanthorrhiza*, SFME-extracted oils had a greater inhibitory effect on the mushroom tyrosinase enzyme than HD-extracted oils, whereas essential oils of *C. aromatica* obtained from either method exhibited almost similar effects on the enzyme. In terms of enzyme-inhibitory action, SFME-extracted oils are comparatively better than HD-extracted oils, which might be due to the higher efficiency of the microwave extractor in the recovery of the bioactive compounds [67]. Similar findings have been reported by Maaiden et al. [68], who reported that microwave-assisted extraction (MAE) exhibited the highest anti-tyrosinase activity compared to maceration and Soxhlet extraction in some medicinal and aromatic plants.

### 2.5. Anti-Cancer Activity

**Figure 7:**
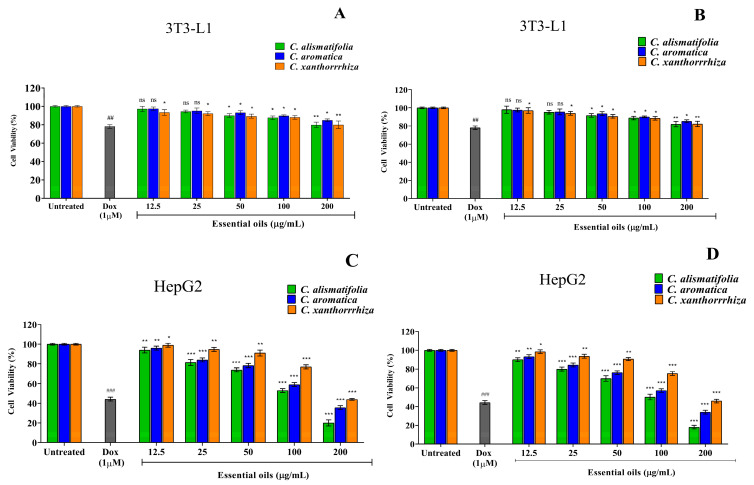
Anticancer activity of *Curcuma* essential oils extracted using HD (**A**,**C**) and SFME (**B**,**D**) methods, showing cell viability on 3T3-L1 non-cancer cells and HepG2 cancer cells using the MTT assay after 24 h. The results of three independent experiments are presented as means ± SD (*n* = 3). ^##^ *p* < 0.01 and ^###^ *p <* 0.001 compared with the untreated group; ^ns^ *p* > 0.05, * *p* < 0.05, ** *p* < 0.01 and *** *p* < 0.001 compared with the untreated group as determined by Dunnett’s multiple comparison test.

**Figure 8:**
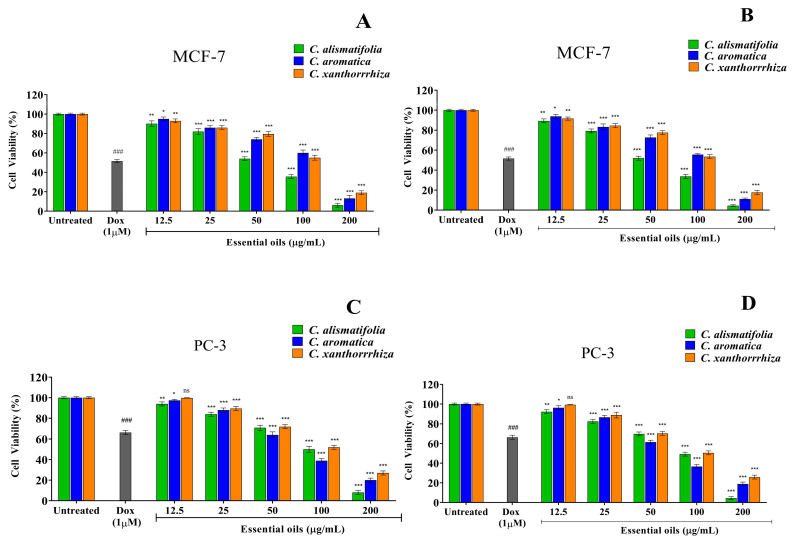
Anticancer activity of *Curcuma* essential oils extracted using HD (**A**,**C**) and SFME (**B**,**D**) methods showing cell viability on MCF-7 and PC-3 cancer cells using the MTT assay after 24 h. The results of three independent experiments are presented as means ± SD (*n* = 3). ^###^ *p <* 0.001 compared with the untreated group; ^ns^ *p* > 0.05, * *p* < 0.05, ** *p* < 0.01 and *** *p* < 0.001 compared with the untreated group as determined by Dunnett’s multiple comparison test.

The standard MTT assay was used to evaluate the anti-proliferative activity of *Curcuma* essential oils against HepG2, MCF7 and PC3 cancerous cell lines. The 3T3-L1 non-cancerous/normal cell line was used to investigate the toxicity of the essential oils. When cells were exposed to increasing doses of essential oil (6.25–200 µg/mL), their viability decreased significantly (Figure 7 and Figure 8). To compare the inhibitory action, doxorubicin was used as the standard reference drug that showed an IC_50_ value of 1.43 ± 0.21 µg/mL, 1.16 ± 0.19 µg/mL, 2.02 ± 0.38 µg/mL and 811 ± 1.7 µg/mL in HepG2, MCF7, PC3 and 3T3-L1 cells, respectively. The inhibitory action of doxorubicin in the current study is somewhat similar to that reported in previous studies [69,70,71]. The MTT assay results suggested that the essential oil of *C. alismatifolia* showed significant selective cytotoxicity against MCF7 and PC3 cells, having an IC_50_ value of 55.62 ± 0.89 µg/mL vs 52.86 ± 1.23 µg/mL for HD vs SFME and 102.24 ± 2.56 µg/mL vs 98.61 ± 1.87 for HD vs SFME, respectively, confirming the anti-breast and anti-prostate cancer potency. *C. aromatica* (71.67 ± 1.89 μg/mL vs. 69.92 ± 1.44 μg/mL for HD vs. SFME) and *C. xanthorrhiza* (97.28 ± 2.81 μg/mL vs. 94.22 ± 1.98 μg/mL for HD vs. SFME) essential oils exhibited promising anti-prostate cancer efficacy against PC3 cells. Additionally, the three *Curcuma* essential oils showed good anticancer properties against HepG2 cells exhibiting medium IC_50_ values (Table 3). *Curcuma* essential oils on 3T3-L1 revealed a non-toxic effect with high IC_50_ values, leading to the conclusion that the essential oils of *Curcuma* are safe to use and do not have any adverse side effects.

According to previous studies, *Curcuma* rhizome and leaf essential oils induce apoptosis or cell death in liver and lung cancer cells [72,73]. By inducing apoptotic cell death in multiple cancer cells, the essential oil of *C. aromatica* was found to have proven anticancer properties [13,74]. *C. xanthorrhiza* is reported to have anticancer properties against different cancer cell lines including cervical, breast, lung, colon, liver, oral, oesophageal and skin cancers due to the presence of xanthorrhizol [75]. This is the first in vitro anticancer study of *C. alismatifolia* essential oil with significant inhibitory ability, thus making it the most effective anticancer agent among the three *Curcuma* species.

Specific compounds like germacrone [76,77], curzerenone [77], camphor [78], 1,8 cineole [79,80], terpinolene [81] and α-pinene [82] which are previously known to cause apoptosis and signalling disruption in cancer cells, might be attributed to the cytotoxicity of essential oils. The anticancer properties of essential oils were not greatly influenced by the extraction process, while SFME-extracted oils were slightly better than HD-extracted oils due to their low IC_50_ values. Very few reports are available on the effect of anticancer properties of oils isolated using different extraction methods, and here too, no correlation between the methods of extraction and anticancer activity could be established [83,84].

## 3. Materials and Methods

### 3.1. Chemicals and Reagents

For the study, 2,2-diphenyl-1-picrylhydrazyl (DPPH), 2,2′-azino-bis (3-ethylbenzothiazoline-6-sulfonic acid) (ABTS), DMEM-Low glucose, DMEM-high glucose, MTT Reagent, Butylated hydroxytoluene (BHT), ascorbic acid and ammonium persulfate were purchased from Hi-Media Pvt. Ltd. (Mumbai, India). Mushroom tyrosinase and doxorubicin were procured from Sigma-Aldrich Chemicals Pvt. Ltd. (Bengaluru, India). L-DOPA and sodium phosphate were obtained from SRL Pvt. Ltd. (Mumbai, India). Kojic acid was ordered from Cayman chemicals (Ann Arbor, MI, USA). Methanol and DMSO used were of analytical grade.

### 3.2. Cell lines and Culture Medium

Human hepatocellular adenocarcinoma (HepG2) cells, human prostate adenocarcinoma (PC3) cells, Human breast adenocarcinoma (MCF7) cells, and Mouse non-malignant embryo fibroblast (3T3-L1) cells were obtained from the Cell Bank of the National Centre for Cell Science (NCCS), Pune, India. The HepG2 cells were maintained in DMEM low-glucose media and PC3, MCF7, 3T3-L1 cells were maintained in DMEM high-glucose media supplemented with 10% FBS (Himedia, Thane, India) along with the 1% antibiotic-antimycotic solution and 1% L-Glutamine (200 mM). All cultures were grown in a humidified 5% CO_2_ incubator at 37 °C and the cells were sub-cultured every two days.

### 3.3. Plant Materials

The rhizomes of three *Curcuma* species were harvested and collected in September 2022 from the net house/greenhouse of the Centre for Biotechnology, Siksha O Anusandhan (Deemed to be University), Bhubaneswar, India (20°17′1.348″ N, 85°46′32.37″ E, 75 m above sea level). The rhizomes were mature and healthy with uniform size and vigour, and were grown under similar pedoclimatic conditions. The species were authenticated and identified by Prof. Pratap Chandra Panda, Taxonomist, and voucher specimens of the three *Curcuma* species were preserved in the Herbarium of the Centre for Biotechnology, Siksha ‘O’ Anusandhan (Deemed to be University), Bhubaneswar, Odisha, India. The following were the voucher specimen numbers: *Curcuma alismatifolia* (CBT/2441), *Curcuma aromatica* (CBT/2442) and *Curcuma xanthorrhiza* (CBT/2443).

### 3.4. Extraction Methods

The essential oils of the three *Curcuma* species were extracted using conventional hydrodistillation (HD) and solvent-free microwave extraction (SFME) methods.

#### 3.4.1. Hydrodistillation (HD)

Dried rhizomes of *Curcuma* species were rehydrated and hydrodistillated for 7 h using a Clevenger apparatus. The volatile oils were then extracted until no more oil was obtained in the apparatus and dried with sodium sulphate (anhydrous) where the yield was calculated as the ratio of essential oil volume to dried rhizome weight (%*v*/*w*). The essential oils were stored in the refrigerator at 4 °C for subsequent analysis. Further, the essential oils were subjected to chemical profiling using a targeted GC-MS analysis and bio-activities.

#### 3.4.2. Solvent Free Microwave Extraction (SFME)

**Figure 9:**
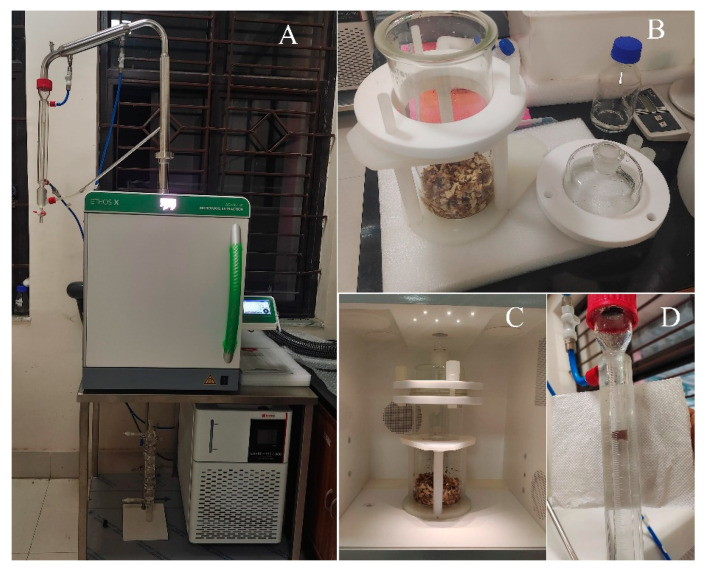
(**A**) Milestone ETHOS X advanced microwave green extraction chamber; (**B**) integrated glass vessel of 2 L capacity and glass lid; (**C**) glass reactor containing sample inside the extraction chamber; (**D**) condensed *Curcuma* essential oil in the burette.

Solvent-free microwave-assisted extraction for three *Curcuma* samples was performed using a Milestone ETHOS X (Sorisole, Italy) advanced microwave extraction device (Figure 9A). An infrared sensor to track the temperature and two magnetrons combined to deliver a maximum power of 1800 W (2 × 950 W) are included in this 2.45 GHz multimode microwave reactor. At atmospheric pressure, the extraction was initiated using an integrated glass vessel /reactor with a capacity of 2 L covered by a glass lid (Figure 9B,C). Dried rhizomes were soaked with water for 30 min within the integrated glass vessel and processed through the system. Power was set at 450 W for the first 30 min, then dropped to 400 W for the rest period. The parameters such as time, pressure, power and temperature were regulated by the instrument’s programme/software. The Fragrances setup was used to configure the system, which consists of a glass Clevenger apparatus placed above the oven, continuously condensing the volatile substances and enabling water to re-enter the reactor. The volatile oils were collected from the apparatus (Figure 9D) and treated with sodium sulphate (anhydrous). The yield was determined as the weight of dried rhizomes divided by the volume of essential oil (%*v*/*w*). Further, the samples were preserved in a refrigerator at 4 °C for subsequent analysis.

### 3.5. Compound Identification Using GC-MS Analysis

Essential oils extracted from both extraction methods were analysed and quantified using a Clarus 580 Gas Chromatograph (Perkin-Elmer, Waltham, MA, USA) associated with an SQ8S mass spectrometric detector. A volume of 0.5 µL was employed for the sample injection. Helium was utilized as the carrier gas in the experiment at a flow rate of 1.0 mL/min and an Elite-5MS capillary column (30 m × 0.25 mm I.D. × 0.25 m thickness) was used. Initially set at 60 °C, the oven’s temperature was increased to 220 °C at a rate of 3 °C/min and then held for 7 min. The ion source temperature was kept at 150 °C, while the injector temperature was kept at 250 °C. The ionization energy was set at 70 eV, and a range of 50 to 600 amu was scanned. The mass spectra of each separated component were compared with the mass spectra of NIST database and retention indices (RI) relative to a homologous series of n-alkane (C_8_–C_20_) on the Elite-5MS capillary column were compared to an existing bibliographic database to identify the constituents [40].

### 3.6. Antioxidant Assay

The antioxidant activity of the three *Curcuma* species was carried out using two different assays: 2,2-diphenyl-1-picrylhydrazyl (DPPH) radical scavenging assay and 2,2ʹ-azinobis-(3-ethylbenzoithazoline-6-sulfonic acid) (ABTS^+^) radical cation-based assay. The samples were diluted using methanol (MeOH) and prepared for various concentrations (1–200 µg/mL).

#### 3.6.1. DPPH Assay

The antioxidant potential of oil was evaluated by DPPH free radical scavenging ability by following the methodology of Chang et al. [85] with slight modifications. The mixture of 1 mL of the various sample concentrations (1 to 50 µg/mL) and 1 mL of the methanolic DPPH solution (0.1 mM) was maintained at room temperature for 30 min. The same protocol was followed for ascorbic acid and butylated hydroxytoluene (BHT) that were taken as the standards. The absorbency of the samples was recorded using a UV-Visible spectrophotometer at 517 nm. The mixture of methanol and DPPH without sample was used as a control. More radical scavenging activity is indicated by a lower degree of absorption. The formula below was used to determine the amount of DPPH free radical inhibition by the essential oils:

Inhibition % = (1 − sample absorbance/control absorbance) × 100(1)

#### 3.6.2. ABTS Assay

The ABTS method described by Kamila et al. [86] was used to evaluate the oil’s antioxidant capacity following some minor modifications. An ABTS reagent was prepared by mixing 2.45 mM ammonium persulfate with 7 mM ABTS solution, then the reagent was incubated in the dark for 16 h at 37 °C. An optical density of 0.70 ± 0.08 at 734 nm should be obtained by diluting the ABTS solution with methanol prior to the experiments. Then, 3 mL of ABTS solution was mixed with 0.3 mL of different sample concentrations (1 to 50 µg/mL). The same methodology was used for the standards, ascorbic acid and butylated hydroxytoluene (BHT). A control solution was prepared using the methanol and ABTS reagent without sample. The absorbance was measured at 734 nm and the following formula was used to determine the % inhibition of the ABTS free radical:

Inhibition % = (absorbance of control − absorbance of sample/absorbance of control) × 100(2)

### 3.7. Anti-Tyrosinase Assay

The tyrosinase inhibitory study was performed with minor modifications to the method reported by Rahmani et al. [87]. L-DOPA (0.5 mg/mL), mushroom tyrosinase (1000 units/mg) and kojic acid were taken as the substrate, enzyme and positive control, respectively. L-DOPA and mushroom tyrosinase were prepared using phosphate buffer (PB) (0.1 M) (pH 6.8) whereas the stock solution for essential oil samples and kojic acid were prepared with 1% DMSO solution since the solubility of oil samples was low in 100% DMSO. The subsequent working concentrations/ dilution (12.5–200 µg/mL) of the oil samples and standard kojic acid were made using the phosphate buffer. In a 96-well plate, each sample consisted of four wells labelled as A, B, C and D, where each well contained a reaction mixture of 180 µL. The following are the contents of each well: A (20 µL enzyme + 140 µL PB), B (20 µL 1% DMSO + 140 µL PB), C (20 µL enzyme + 140 µL PB + 20 µL sample) and D (140 µL PB + 20 µL sample). The reaction mixture was properly mixed and incubated at 25 °C for 10–15 min. After the incubation period, 20 µL of L-DOPA was added to all wells (A, B, C and D) and incubated at 25 °C for 20 min. Then, the absorbency of the samples was noted using EPOCH 2 microplate spectrophotometer (Agilent BioTek) at 475 nm. Using the following formula, the amount of tyrosinase inhibition was calculated; Inhibition % = 100 [(A − B) − (C − D)]/(A − B), where A = The change in absorbance before and after incubation without sample, B = The change in absorbance before and after incubation without sample and enzyme, C = The change in absorbance before and after incubation with oil sample, D = The change in absorbance before and after incubation with oil sample but without enzyme. The experiment was conducted in triplicate.

### 3.8. Anti-Cancer Activity

The anticancer activity of the essential oils was evaluated against MCF7, HepG2, and PC3 cancer cells by measuring the rate of cell proliferation using the colorimetric MTT [3-(4,5-dimethyl-2-thiazolyl)-2,5-diphenyltetrazolium bromide] assay [88,89]. Cells were seeded at a density of 2 × 10^4^ cells/well on a 96-well plate and 20 μL of essential oils in were added in various concentrations (6.25–200 µg/mL) to the microplates and incubated for 24 h at 37 °C in a 5% CO_2_ atmosphere. After discarding the used media, MTT reagent was added at a final concentration of 0.5 mg/mL of the overall volume, and the mixture was then incubated in a CO_2_ incubator at 37 °C for 3–4 h. The supernatants were then aspirated and 100 μL of solubilization solution (DMSO) was added to dissolve the formed formazan crystals. The absorbance was measured using an ELISA reader at 570 nm. DMSO and doxorubicin were taken as control and standard, respectively. Percentage of cell viability was calculated using the formula, % Cell viability = [(A_a_ − A_b_)/(A_c_ − A_b_)] × 100, Where A_a_ = Absorbance value of sample (essential oil), A_b_ = Absorbance value of blank and A_c_ = Absorbance value of the control. The IC_50_ value of the MTT experiment was defined as the concentration of essential oil that resulted in 50% cytotoxicity and was determined by using a linear or logarithmic regression equation i.e., Y = Mx + C where, Y = 50, M and C values were derived from the regression equation.

### 3.9. Statistical Analysis

The outcomes of independent experiment were performed in triplicates and were expressed as mean ± SD. To analyse the statistical differences in EO yield (%) and quality across *Curcuma* species, a one-way ANOVA followed by a post-hoc test was carried out using the statistical software (Minitab 17). The data were also analysed using OriginPro (2023 version), GraphPad Prism 8.0.2 and Microsoft Office Excel (version 2304, 2019).

## 4. Conclusions

The standard hydro-distillation (HD) method has been compared with the solvent-free microwave extraction (SFME) technique for the extraction and bioactivity study of essential oils from three different *Curcuma* species viz. *C. alismatifolia*, *C. aromatica* and *C. xanthorrhiza*. The SFME method proved to be significantly advantageous over the conventional HD method in terms of oil yield, extraction time, energy saving, solvent (water) consumption and waste generation. A similar qualitative profile of essential oils of three studied species was revealed by GC-MS, while quantitative variation between the major compounds was detected in the chemical composition using the two extraction methods. A total of 85 compounds were identified among the three *Curcuma* species, with hydrocarbons and oxygenated rich compounds predominating the essential oils extracted using HD and SFME, respectively. Essential oils extracted through HD and SFME were shown to have strong antioxidant, anti-tyrosinase and anticancer properties, with *C. alismatifolia* essential oil having most the potent bioactivity. The essential oil extracted by SFME was found to have greater antioxidant, anti-tyrosinase, and anticancer activities than those isolated employing the HD method. The current results suggest that SFME-generated essential oil might be a valuable source of bioactive compounds and constitute potential antioxidant, anti-tyrosinase and anticancer agents for application in food, health and cosmetic products.

## Figures and Tables

**Table 1:** Comparison of the two extraction methods for each *Curcuma* species in terms of extraction time, energy consumption, essential oil colour and capacity for hydration.

Samples	Extraction Time (h)		Energy Consumption (kWh)		Colour of Essential Oil		Water (mL/100 g)	
	HD	SFME	HD	SFME	HD	SFME	HD	SFME
*C. alismatifolia*	7	4	9.43	2.13	Pale Yellow	Deep Yellow	1000	100
*C. aromatica*	7	4	9.43	2.13	Deep brown	Light brown	1000	100
*C. xanthorrhiza*	7	4	9.43	2.13	Pale yellow	Pale yellow	1000	100

**Table 3:** Cytotoxic effects (IC_50_) of *Curcuma* essential oils on growth of various cell lines as determined by MTT assay.

Samples			IC_50_ Value (µg/mL)					
	MCF7		HepG2		PC3		3T3-L1	
	HD	SFME	HD	SFME	HD	SFME	HD	SFME
*C. alismatifolia*	55.62 ± 0.89 ^a^	52.86 ± 1.23 ^b^	111.89 ± 2.45 ^a^	108.97 ± 2.10 ^b^	102.24 ± 2.56 ^a^	98.61 ± 1.87 ^b^	540.62 ± 4.32 ^b^	551.23 ± 5.12 ^a^
*C. aromatica*	115.89 ± 1.83 ^a^	108.87 ± 1.07 ^b^	140.58 ± 1.04 ^a^	139.41 ± 1.61 ^a^	71.67 ± 1.89 ^a^	69.92 ± 1.44 ^a^	745.89 ± 9.10 ^b^	761.04 ± 8.32 ^a^
*C. xanthorrhiza*	129.78 ± 1.92 ^a^	115.57 ± 1.58 ^b^	191.49 ± 1.48 ^a^	187.94 ± 1.78 ^b^	97.28 ± 2.81 ^a^	94.22 ± 1.98 ^b^	626.54 ± 8.90 ^b^	639.48 ± 5.88 ^a^

Data are represented as mean ± S.D (*n* = 3). Distinct letters in each row signify a significant difference in the mean value at *p* < 0.05.

## Data Availability

The data presented in this work are available in the article.

## Sample Availability

Samples of the compounds are not available from the authors.
